# Medical cannabis for the treatment of comorbid symptoms in children with autism spectrum disorder: An interim analysis of biochemical safety

**DOI:** 10.3389/fphar.2022.977484

**Published:** 2022-09-29

**Authors:** Orit Stolar, Ariela Hazan, Roni Enten Vissoker, Ibrahim Abu Kishk, Dana Barchel, Mirit Lezinger, Adi Dagan, Nir Treves, David Meiri, Matitiahu Berkovitch, Elkana Kohn, Eli Heyman

**Affiliations:** ^1^ Autism Center/ALUT, Shamir (Assaf Harofeh) Medical Center, Affiliated to Sackler School of Medicine, Tel-Aviv University, Tel-Aviv, Zerifin; ^2^ Clinical Pharmacology and Toxicology Unit, Shamir (Assaf Harofeh) Medical Center, Affiliated to Sackler School of Medicine, Tel-Aviv University, Tel-Aviv, Zerifin; ^3^ Pediatric Neurology Department, Shamir (Assaf Harofeh) Medical Center, Affiliated to Sackler School of Medicine, Tel-Aviv University, Tel-Aviv, Zerifin; ^4^ Division of Clinical Pharmacy, Institute for Drug Research, School of Pharmacy, Faculty of Medicine, The Hebrew University of Jerusalem, Jerusalem, Israel; ^5^ Technion—Israel Institute of Technology, Haifa, Israel

**Keywords:** biochemical safety, cannabis, medical, autism spectrum, disorder, pediatric

## Abstract

**Background:** Autistic Spectrum Disorder (ASD) is a common neurodevelopmental disorder and no effective treatment for the core symptoms is currently available. The present study is part of a larger clinical trial assessing the effects of cannabis oil on autism co-morbidities.

**Objectives:** The aim of the present study was to assess the safety of a CBD-rich oil treatment in children and adolescents with ASD.

**Methods:** Data from 59 children and young adults (ages 5–25 years) from a single-arm, ongoing, prospective, open-label, one center, phase III study was analyzed. Participants received the Nitzan Spectrum® Oil, with cannabis extracts infused in medium chain triglyceride (MCT) oil with a cannabidiol:THC ratio of 20:1, for 6 months. Blood analysis was performed before treatment initiation, and after 3 months. Complete blood count, glucose, urea, creatinine, electrolytes, liver enzymes (AST, ALT, gamma glutamyl transferase), bilirubin, lipid profile, TSH, FT4, thyroid antibodies, prolactin, and testosterone measurements were performed at baseline, prior to starting treatment and at study midpoint, after 3 months of treatment.

**Results:** 59 children (85% male and 15% female) were followed for 18 ± 8 weeks (mean ±SD). The mean total daily dose was 7.88 ± 4.24 mg/kg body weight. No clinically significant differences were found in any of the analytes between baseline and 3 months follow up. Lactate dehydrogenase was significantly higher before treatment (505.36 ± 95.1 IU/l) as compared to its level after 3 months of treatment (470.55 ± 84.22 IU/L) (*p* = 0.003). FT4 was significantly higher after 3 months of treatment (15.54 ± 1.9) as compared to its level before treatment (15.07 ± 1.88) (*p* = 0.03), as was TSH [(2.34 ± 1.17) and (2.05 ± 1.02)] before and after 3 months of treatment, respectively (*p* = 0.01). However, all these values were within normal range. A comparison of the group with additional medications (*n* = 14) to those who received solely medical cannabis (*n* = 45) showed no difference in biochemical analysis, including liver enzymes, which remained stable, except for change in potassium level which was significantly higher in the group that did not receive additional medications (0.04 ± 0.37) compared to the group receiving concomitant drug therapy (-0.2 ± 0.33) (*p* = 0.04). A comparison of patients who received a high dose of the cannabis oil (upper quartile-16 patients), with those receiving a low dose (lower quartile—14 patients) showed no significant difference between the two groups, except for the mean change of total protein, which was significantly higher among patients receiving high dose of CBD (0.19 ± 2.74) compared to those receiving a low dose of CBD (1.71 ± 2.46 (*p* = 0.01), and mean change in number of platelets, that was significantly lower among patients who received high dose of CBD (13.46 ± 31.38) as compared to those who received low dose of CBD (29.64 ± 26.2) (*p* = 0.0007). However, both of these changes lack clinical significance.

**Conclusion:** CBD-rich cannabis oil (CBD: THC 20:1), appears to have a good safety profile. Long-term monitoring with a larger number of participants is warranted.

## 1 Introduction

Autistic Spectrum Disorder (ASD) is a neurodevelopmental disorder that includes a wide range of complex developmental disabilities. The core symptoms of autism include impairments in social interaction and communication, as well as the presence of restricted and repetitive behaviors (APA, 2013). Despite its prevalence among the common neuro-biological-based childhood disorders, no effective treatment for the core symptoms of ASD is currently available, and to date, the standard of care consists primarily of behavioral interventions (Zamberletti et al., 2017). The only two medications currently approved by the FDA for the indication of irritability in children with ASD are risperidone and aripiprazole. In clinical practice, physicians often prescribe other drugs such as SSRI’s and methylphenidate off label, to treat the behavioral and social deficit related co-morbidities of ASD, and the effects of these medications are inconsistent (Lai et al., 2014).

One of the newer treatment options being investigated for ASD, as well as an array of other conditions, is cannabidiol (CBD)-rich cannabis extract (Barchel et al., 2019; Aran and Rand, 2020; Ponton et al., 2020). Conditions such as anorexia, emesis, pain, inflammation, multiple sclerosis, neurodegenerative disorders (Parkinson’s disease, Huntington’s disease, Tourette’s syndrome, Alzheimer’s disease), epilepsy, glaucoma, osteoporosis, schizophrenia, cardiovascular disorders, cancer, obesity, and metabolic syndrome-related disorders, to name a few, are also being treated or have the potential to be treated by cannabinoid agonists/antagonists/cannabinoid-related compounds (Schlag, 2020).

Cannabis contains a number of active compounds, including Δ9-tetrahydrocannabinol (Δ9-THC), cannabidiol (CBD) and terpenoids (Russo, 2011). CBD has anti-emetic, anti-convulsive, anti-psychotic, anti-inflammatory, antioxidant and neuroprotective properties (Cheng et al., 2014) and is generally well-tolerated (Devinsky et al., 2014; Detyniecki and Hirsch, 2015). Δ9-Tetrahydrocannabinol (THC) has psychoactive and relaxant effects and often imparts a sense of euphoria, a quality which underlies its utility as a treatment for pain and nausea. Δ9-THC also activates the endocannabinoid system in the central nervous system and affects anxiety, appetite, cognitive function and memory (Palmieri et al., 2017).

Both CBD and THC bind to both the CB1/CB2 receptors in the human endocannabinoid system (ECS), with varying levels of affinity (Gallily et al., 2015).

The human endocannabinoid system, composed of endogenous, lipid-based retrograde neurotransmitters that bind to cannabinoid receptors and cannabinoid receptor proteins throughout the central and peripheral nervous system (Freitas et al., 2018) is often affected in conditions such as epilepsy, anxiety, cognitive impairments and sleep pattern disturbances (Zamberletti et al., 2017). The ECS has drawn attention in recent years as a potential contributor to ASD, due to its role in regulation of synaptic function through its inhibition of the release of neurotransmitters from presynaptic neurons (Ponton et al., 2020).

The majority of the available data on the safe use of CBD in children is on the treatment of epilepsy, specifically FDA-approved Epidiolex® (Devinsky et al., 2017). Recently, increasing preclinical and clinical data have highlighted the therapeutic benefits of cannabinoids for individuals with ASD. Reviews which have examined the effects of cannabis types and dosages on common co-morbidities in ASD (including irritability, hyperactivity, sleep disorders, self-injurious behavior, anxiety) describe positive results (Barchel et al., 2019). Yet, despite evidence for its efficacy, concerns about adverse outcomes, such as agitation, somnolence, decreased appetite, and irritability have been raised (Agarwal et al., 2019; Poleg et al., 2019). In addition, data on the increased risk of hepatotoxicity, mainly when co-administered with valproate, have accentuated the need for further study of its biochemical safety (Samanta, 2019; Sands et al., 2019; Aran and Rand, 2020; Lattanzi et al., 2020).

The effect of medical cannabis on hormones such as prolactin, thyroid function and testosterone, should be is an under-explored area of study. In one double-blind study of 11 healthy adults, in which CBD or a placebo at doses of 300 mg or 600 mg were administered by injection, basal prolactin and growth hormone levels remained unchanged both after placebo and CBD. We have yet to identify literature which suggests CBD has any adverse effect on kidney function (Rein, 2020). As a result, the goal of the current study was to assess the biochemical safety of CBD-rich cannabis oil in children with ASD.

## 2 Aim and study population

The aim of this prospective cohort was to assess the safety-related blood tests of children and young adults with ASD taking a CBD-rich cannabis oil-based product. Participants were recruited during the period spanning November 14 2019 to May 25 2021. Data from a baseline sample (pre-treatment) of 59 children and young adults (ages 5–25 years) from an ongoing prospective single-arm, open-label, one center, phase 3 study was analyzed. Eligibility for inclusion in the study included a diagnosis of ASD (DSM-5) by a pediatric neurologist or a pediatric psychiatrist in the community healthcare setting. In addition, patients were required to have at least one severe co-morbidity, such as problems with sleep, aggression/self-injury behaviors, anxiety, or irritability that existed for at least 6 months. Prior to enrollment, treatment and collection of data, written informed consent was obtained from the parents of all participants. Children and adolescents with a known genetic syndrome such as tuberous sclerosis, Fragile X syndrome, and Angelman syndrome were excluded, as were those currently receiving or who had received medical cannabis therapy in the past, had current psychosis, schizophrenia or schizo-affective disorder or had a first degree relative with these disorders. Children diagnosed with a metabolic illness, immune disorder or liver cancer, or epilepsy with clinical symptoms (presence of non-clinical epileptic activity did not represent a reason for exclusion) were also excluded from participating. Ethical approval for the study was obtained from the local Helsinki committee (Number 281–17-ASF) and was registered with ClinicalTrials.gov (NCT05212493).

## 3 Methods

### 3.1 Treatment protocol

The treatment protocol was individualized for each patient using a personalized medicine approach. Participants received medical cannabis extract infused in MCT oil with a CBD:THC ratio of 20:1 (Or Nitzan Spectrum®, Seach Medical Group, Israel, manufactured by Nextage Therapeutics, Israel) for 6 months. Parents were instructed to start with one oral drop daily (each drop contains: 0.3 mg THC and 5.7 mg CBD) and increase the dosage gradually until they perceived improvements in their child’s behavior such as decreased irritability, aggressiveness, hyperactivity, and/or sleep disturbances. The amount and timing of doses during each day was tailored to individual needs of the child (e.g., higher dose at night if needed for sleep support). Parents completed a bi-weekly phone interview where they reported compliance, behavior, symptoms, and side effects. The maximum dose did not exceed 10 mg/kg/day (or total of 400 mg/day) of CBD and 0.5 mg/kg/day (or total of 20 mg/day) of THC based upon previous findings (Aran and Rand, 2020). Information on comorbid symptoms and safety was recorded biweekly during follow-up interviews during the 6 month study. Participants taking any medications prior to entering the study were instructed not to make any changes during the study period.

### 3.2 Primary and secondary outcome measures

Medical interview was performed and weight/height measurements collected. Primary outcome measures included blood analytes which included: complete blood count, glucose, urea, creatinine, electrolytes, liver enzymes (aspartate aminotransferase (AST), alanine aminotransferase (ALT), gamma glutamyl transferase (GGT)), bilirubin, lipid profile, thyroid stimulating hormone (TSH), free T4, thyroid antibodies, prolactin, and testosterone. CBC was measured in whole blood and all other biochemistry parameters were measured in the serum using Cobas® 8000 analyzer (Roche Diagnostics). Blood tests were performed in the morning, at baseline, prior to starting treatment and at study midpoint, after 3 months of treatment. Secondary outcomes were change in safety tests both with varying doses of CBD as well as with concomitant drug usage.

### 3.3 Study procedure

Baseline–All participants underwent a medical history and physical exam, completed questionnaires and underwent blood testing, growth measurements, EEG, Autism Diagnostic Observation Schedule (ADOS), Vineland adaptive behavior scales, and cognitive tests.

Follow up—Biweekly during follow-up interviews by telephone were conducted throughout the study. At midpoint (3 months), an in person interview was conducted and blood work and growth indices were taken. After 6 months, growth measurements, ADOS, EEG, Cognitive and Vineland adaptive behavior scales were completed.

### 3.4 Statistical analysis

Data is presented as mean and standard deviation for continuous variables and percentage of frequency for categorical variables. Blood count and biochemical blood tests were tested before and after treatment using paired *t*-test. Unpaired *t*-test was utilized in two secondary analyses. The first analysis examined the differences before and after CBD treatment in patients treated with co-medications versus patients who were not exposed to other medications; the second analysis compared the differences before and after treatment in patients exposed to high CBD dosage (dose belongs to the upper quartile, i.e. 3.51–6.53 mg/kg per day) versus patients exposed to low CBD dosage (dose belongs to the lowest quartile, i.e. 0.71–1.78 mg/kg per day). Data was analyzed with “PANDAS” and “Scipy“ packages in python via the platform of Jupyter notebook. The significance levels were set at 0.05.(Mckinney, 2010; Kluyver et al., 2016).

## 4 Results

59 children (85% males and 15% females) were followed for 18 ± 8 weeks (mean ±SD).

Baseline patient characteristics are presented in Table 1.

**TABLE 1:** Patients characteristics and baseline symptoms.

Characteristics		
Sex, n (%)	Male	50 (84.7)
	Female	9 (15.3)
Age (years), mean (STD, range)		10.7 (4.6, 5–25)
Concomitant drugs, n (%)	No	45 (76.2)
	Yes	14 (23.8)
Medications, n (%)	Stimulants	6 (10.2)
	Typical antipsychotics	2 (3.4)
	Atypical antipsychotics	6 (10.2)
	Anti-epileptic	2 (3.4)
	Melatonin	5 (8.5)
	Anti-depressant	2 (3.4)
	Other anti-muscarinic	1 (1.7)
	Alpha agonist	1 (1.7)
Mean CBD Daily dose per mg/kg		2.75 (1.30)

The average doses in the morning, noon, evening and the daily total doses were as follows: Morning dose mean—4.04 ± 1.71 drops body weight; Noon mean—2.02 ± 2.28; Evening mean—3.03 ± 1.84 and Total day—7.88 ± 4.24 drops body weight (amount and timing of doses during each day was tailored to individual needs of the child, as mentioned above (e.g., higher dose at night if needed for sleep support)).

### 4.1 Biochemical analysis

No clinical or statistically significant differences were found in any of the analytes between baseline and at 3 months follow up. No significant change was observed in complete blood count, including hemoglobin, red blood cells or leucocytes, before and after 3 months of treatment. Platelet levels were higher before treatment (1.63 ± 46.75) compared to after treatment (8.08 ± 26.68) (*p* = 0.5) however those values are all within the normal range.

No statistically significant difference was observed in urea or creatinine before or after 3 months of treatment. No statistically significant difference was observed in liver enzymes (AST, ALT, ALP), thyroid hormones, thyroid antibodies, prolactin, or other hormones (Table 2). All of these values were within normal range. LDH was significantly higher before treatment (505.36 ± 95.1 IU/l)) as compared to its level after 3 months of treatment (470.55 ± 84.22 IU/L) (*p* = 0.003). Upon further examination of thyroid function measures, it appears that TSH values were tending to decrease while FT4 tended to increase. However, the changes in both measures remained within normal ranges. FT4 was significantly higher after 3 months of treatment (15.54 ± 1.9) as compared to its level before treatment (15.07 ± 1.88) (*p* = 0.03). TSH was higher before treatment (2.34 ± 1.17) as compared to the level after 3 months of treatment (2.05 ± 1.02) (*p* = 0.01).

**TABLE 2:** Blood analysis.

Test	Pre-treatment (Mean±SD)	After 3 months (Mean±SD)	*p* value
Albumin (ALB) (38–54 g/L)	46.37 ± 4.03	46.95 ± 2.37	0.35
Alkaline phosphatase (ALP) (117–390 U/L)	217.97 ± 71.04	220.79 ± 81.3	0.47
Alanine aminotransferase (ALT) (4–39 U/L)	16.22 ± 6.31	15.36 ± 6.49	0.15
Aspartate aminotransferase (AST) (5–38 U/L)	25.88 ± 6.39	24.88 ± 6.1	0.64
Cholesterol (140–200 mg/dl)	152.24 ± 28.27	125.75 ± 24.2	0.85
Creatine Kinase (CK) (10–170 U/L)	128 ± 68.36	123.47 ± 52.94	0.54
Calcium (Ca) (8.8–10.8 mg/dl)	9.74 ± 0.34	9.76 ± 0.32	0.8
Chloride (Cl) (96–106 nmol/dl)	101.24 ± 2.75	101.55 ± 2.35	0.63
Iron (Fe) (30–110 mcg/dL)	85.55 ± 41.18	83.76 ± 31.04	0.78
Glucose (Glu) (60–100 mg/dl)	88.93 ± 19.05	88.92 ± 16.56	1
Potassium (K) (3.10–5.10 nmol/L)	4.41 ± 0.3	4.43 ± 0.36	0.73
Lactate dehydrogenase (LDH) (240–600 U/L)	505.36 ± 95.1	470.55 ± 84.22	0.003
Sodium (Na) (135–145 nmol/L)	139.46 ± 1.99	139 ± 2.04	0.16
Prolactin (PRL) 4–15.2 (male) 4.8–23.3 (female) µl/L	9.72 ± 8.25	9.72 ± 7.95	0.99
Total protein (PROT-T) (60–80 g/L)	70.82 ± 3.47	70.79 ± 3.13	0.95
Triglycerides (TG) (30–130 mg/dl)	96.27 ± 53.77	94.96 ± 67.79	0.89
Transferrin (TRF) (2–3.6 g/L)	2.83 ± 0.59	2.85 ± 0.41	0.87
Transferrin saturation (TRFsat) (%)	22.85 ± 10.37	22.49 ± 8.91	0.89
Urea (20–45 mg/dl)	27.41 ± 9.9	25.98 ± 7.63	0.24
Creatinine (CR) 0.40–0.60 mg/dl	0.49 ± 0.18	0.49 ± 0.16	0.8
Free T4 (FT4) (12.5–21.5 pmol/L)	15.07 ± 1.88	15.54 ± 1.9	0.03
Thyroid stimulating hormone (TSH) (0.6–4.84 mU/L)	2.34 ± 1.17	2.05 ± 1.02	0.01
Hematocrit (HCT) (40–52%)	40.39 ± 3.06	40.36 ± 2.83	0.9
Platelets (PLT) (0.22–0.3 1000/µl)	283.89 ± 69.41	284.45 ± 70.22	0.92
White blood cells (WBC) (4–11%)	7.79 ± 2.08	7.36 ± 2.1	0.09
Hemoglobin (HGB) (13.5–17.5 g/dl)	13.61 ± 1.14	13.58 ± 1.04	0.75
Testosterone 9.4–37 (male) 0.2–3 (female) nmol/L	2.45 ± 5.11	3.31 ± 6.39	0.1

**FIGURE 1:**
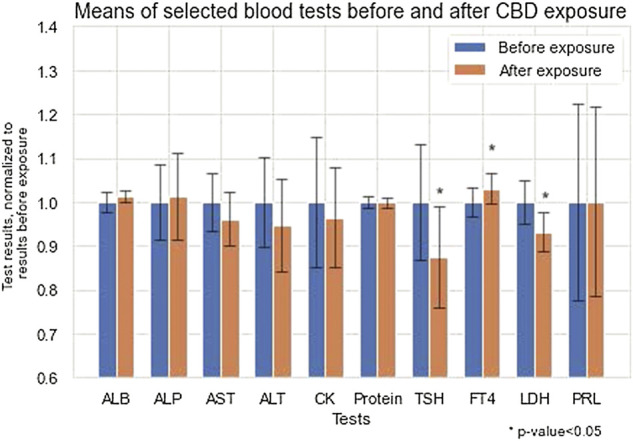
Means of selected bloods tests before and after CBD exposure.

A comparison of the group with additional medications (*n* = 14, Methylphenidate-4, Aripiprazole- 4, Melatonin—3, Risperdal 1, Roxetine-1, Seroquel-1), and those who received solely medical cannabis (*n* = 45) showed no difference in biochemical analysis, including liver enzymes, which remained stable, except of change in potassium level, which was significantly higher before treatment (0.04 ± 0.37) as compared to after 3 months of treatment (-0.2 ± 0.33) (*p* = 0.04). (Supplementary Table S1). We also compared patients who received a high dose of the cannabis oil (upper quartile-16 patients receiving CBD 3.49—6.53 mg/kg body weight), with those receiving low dose (lower quartile - 14 patients receiving CBD 0.7–1/- mg/kg body weight). No significant differences were observed between these two groups with regard to biochemical analysis, except of the mean change of total protein which was significantly higher among patients with high dose of CBD (0.19 ± 2.74) as compared to those with low dose of CBD (1.71 ± 2.46 (*p* = 0.01), and the mean change in number of platelets that was significantly lower among patients who received high dose of CBD (13.46 ± 31.38) as compared to those who received low dose of CBD (29.64 ± 26.2) (*p* = 0.0007) (Supplementary Table S2). The means of selected blood tests before and after CBD exposure is presented in Figure 1.

## 5 Discussion

This study may support the long-term safety of medical cannabis use in children and young adults with ASD. Over the first 3 months of the study, a few statistically significant changes were observed including the level of LDH and thyroid hormones, however, these changes have no clinical significance. The remaining tests which assessed hematological, chemical, and endocrine function, were all within the normal range. In addition, no differences in safety test results were identified between children taking or not taking anti-psychotic medications together with CBD-rich cannabis oil, nor between those receiving high or low dose of medical cannabis.

The recent literature presents a favorable safety profile of CBD in human trials. The majority of the available data comes from research on Epidiolex®, a 100% CBD product, in patients with epilepsy. A 2018 meta-analysis of four randomized controlled trials with patients with Lennox-Gastaut or Dravet syndrome showed that Epidiolex® was associated with a higher rate of increased serum aminotransferases compared to placebo (Lattanzi et al., 2018). Thiele et al. (2019) reported similar findings in their review of young patients with Lennox-Gastaut syndrome, treated with long-term CBD, including increased alanine aminotransferase (ALT) and/or aspartate aminotransferase (AST) in five and four patients, respectively (Thiele et al., 2019).

In a 2020 study on adults given 1500 mg of CBD, in the first 2–4 weeks after initiating treatment, seven (44%) participants experienced peak serum ALT values greater than the upper limit of normal. For five (31%) participants, the value exceeded five times the upper limit of normal, meeting international criteria for drug-induced liver injury (no correlation was identified between transaminase elevations and baseline characteristics, CYP2C19 genotype, or CBD plasma concentrations) (Watkins et al., 2021). Interestingly, in another trial examining the withdrawal effects of adults from CBD (20 mg/kg/day for 4 weeks), the most common adverse event was diarrhea (Taylor et al., 2020). Finally, in a study of two CBD enriched 5% oils (one with 0.25% THC 20:1, the other with 0.83% THC 6:1) on 25 patients with complex motor disorder, no changes in blood tests were found; only three patients experienced elevated CPK by the study’s end and abnormalities of aminotransferase levels were found in one patient only before the study, with no changes identified during the study period (Libzon et al., 2018).

There was no effect on liver function when cannabis was given with other medications, as was reported in previous studies on Epidiolex®. A possible explanation may be that our participants received a lower dosage of phytocannaboids and the medications they received did not have the same effect as was seen with Valporal in the Epidiolex® study. Although a significant change in the TSH, FT4, and LDH was observed, they were all within normal range.

Several studies have reported on the pharmacokinetics and pharmacodynamics of cannabinoids. Two systematic reviews (Lucas et al., 2018; Millar et al., 2018) reported on the pharmacokinetics and pharmacodynamics of cannabidiol using various routes of administration, however, with paucity information in pediatric patients. In a study on healthy beagles which looked at clinical chemistry after administration of a CBD-predominant oil formulation, hematological parameters were generally normal for the dogs across these groups at 1 day and 7 days after the final dose (Vaughn et al., 2020). In their double-blind trial, Zuardi et al. investigated the effects of CBD on plasma prolactin and growth hormone among volunteers who received placebo or oral CBD at the doses of 300 mg or 600 mg; basal prolactin and growth hormone levels were unchanged after CBD (Zuardi et al., 1993).

Although our study was open-label with no placebo group, the findings support the biochemical safety of the preparation used in the study. In order to further evaluate the safety of the preparation used, a comparison was made between patients who were at the upper quartile of cannabis dosage to patients who were at the lower quartile, and no clinical significant difference was observed (Supplementary Table S2).

We also compared biochemistry analysis between patients who received other medications such as risperidone, methylphenidate, melatonin to those who received medical cannabis only (Supplementary Table S1), and the sole finding of a statistically significant change, in potassium levels between these groups, has no clinical significance.

A comparison of patients who received a high dose of the cannabis oil (upper quartile-16 patients receiving CBD 3.49—6.53 mg/kg body weight), with those receiving low dose (lower quartile—14 patients receiving CBD 0.7–1/- mg/kg body weight) showed no significant differences between these two groups with regard to biochemical analysis, except of the mean change of mean total protein, which was significantly higher among patients with high dose of CBD (0.19 ± 2.74) as compared to those with low dose of CBD (1.71 ± 2.46 (*p* = 0.01), and the mean change in number of platelets that was significantly lower among patients who received high dose of CBD (13.46 ± 31.38) as compared to those who received low dose of CBD (29.64 ± 26.2) (*p* = 0.0007) (Supplementary Table S2). However, all of these changes lack clinical significance.

It is possible, however, that due to the small number of patients receiving concomitant medications, no difference was observed.

A theoretical limitation of our study might be the open-label method, however, the biochemistry parameters examined in our study are objective numbers which are not dependent on a double-blind study.

## 6 Conclusion

CBD-rich cannabis oil (CBD: THC 20:1), as part of a monitored treatment program over 3 months, appears to have a good safety profile. Long-term monitoring with larger number of participants is needed. Al-Beltagi, 2021.

## Data Availability

The raw data supporting the conclusion of this article will be made available by the authors, without undue reservation.
