# Supplementary material for: Medical cannabis for the treatment of comorbid symptoms in children with autism spectrum disorder: An interim analysis of biochemical safety
Source: Front Pharmacol. 2022 Sep 29;13:977484. doi: 10.3389/fphar.2022.977484 (PMC9559854; doi:10.3389/fphar.2022.977484)
Supplement: Supplementary file 1 [file DataSheet1.docx]

**Supplementary tables:**

Supplementary table 1: Changes in blood tests before and after CBD exposure in patients with no additional drugs vs. concomitant therapy

| Test | No additional drugs | Concomitant therapy | | P-value | | |  |
| --- | --- | --- | --- | --- | --- | --- | --- |
|  | Mean change ± STD between pretreatment and after 3 months | N | Mean change ± STD between pretreatment and after 3 months | | N |  | |
| Albumin (ALB) | -0.58±5.22 | 45 | -0.57±2.1 | | 14 | 1.0 | |
| Alkaline phosphatase (ALP) | 1.05±23.94 | 44 | -15±42.21 | | 14 | 0.08 | |
| Alanine aminotransferase (ALT) | 0.75±4.86 | 44 | 1.21±3.53 | | 14 | 0.74 | |
| Aspartate aminotransferase (AST) | 1±4.23 | 44 | 1±3.51 | | 14 | 1.0 | |
| Cholesterol | -0.95±19.6 | 39 | 0.92±17.7 | | 12 | 0.77 | |
| Creatine Kinase (CK) | 8.8±51.63 | 40 | -8.62±57.03 | | 13 | 0.31 | |
| Calcium (Ca) | -0.01±0.45 | 45 | -0.03±0.3 | | 13 | -0.87 | |
| Chloride (Cl) | -0.41±3.69 | 22 | 0±2.71 | | 7 | 0.79 | |
| Iron (Fe) | -0.17±46.6 | 44 | 7.96±51.52 | | 14 | 0.58 | |
| Glucose (Glu) | -0.22±29.28 | 45 | 0.79±12.79 | | 14 | 0.9 | |
| **Potassium (K)** | 0.04±0.37 | **44** | -0.2±0.33 | | **14** | **0.04** | |
| Lactate dehydrogenase (LDH) | 32.33±79.61 | 42 | 42.21±101.84 | | 14 | 0.71 | |
| Sodium (Na) | 0.69±2.58 | 45 | -0.29±2.09 | | 14 | 0.2 | |
| Prolactin (PRL) | 0.3±3.68 | 45 | -1.06±4.61 | | 13 | 0.27 | |
| Total protein (PROT-T) | -0.02±4.35 | 45 | 0.21±3.42 | | 14 | 0.86 | |
| Triglycerides (TG) | -8.23±59.8 | 39 | 32.33±77.4 | | 12 | 0.06 | |
| Transferrin (TRF) | -0.07±0.43 | 14 | 0.14±0.37 | | 5 | 0.35 | |
| Transferrin saturation (TRFsat) | 1.83±12.37 | 14 | -3.76±5.06 | | 5 | 0.35 | |
| Urea | 0.62±10.16 | 45 | 4.04±4.84 | | 14 | 0.23 | |
| Creatinine (CR) | 0±0.07 | 43 | -0.02±0.05 | | 13 | 0.16 | |
| Free T4 (FT4) | -0.4±1.54 | 41 | -0.66±1.53 | | 13 | 0.6 | |
| Hematocrit (HCT) | 0.01±1.76 | 41 | 0.11±2.15 | | 12 | 0.87 | |
| Platelets (PLT) | 1.63±46.75 | 41 | -8.08±26.68 | | 12 | 0.5 | |
| Thyroid stimulating hormone (TSH) | 0.35±0.86 | 45 | 0.09±0.9 | | 13 | 0.35 | |
| White blood cells (WBC) | 0.63±1.71 | 41 | -0.22±2.27 | | 12 | 0.17 | |
| Hemoglobin (HGB) | 0±0.54 | 41 | 0.12±0.78 | | 12 | 0.55 | |
| Testosterone | -0.58±5.22 | 45 | -0.57±2.1 | | 14 | 1.0 | |

Supplementary table 2: The changes in blood tests before and after CBD exposure in patients treated with high CBD dosage vs. low CBD dosage

| Test | High CBD dosage (3.51-6.53 mg/kg per day) | Low CBD dosage (0.71-1.78 mg/kg per day) | | P-value | | |  |
| --- | --- | --- | --- | --- | --- | --- | --- |
|  | Mean change ± STD between pretreatment and after 3 months | N | Mean change ± STD between pretreatment and after 3 months | | N |  | |
| Albumin (ALB) | 0±2.66 | 16 | 0.93±2.43 | | 14 | 0.33 | |
| Alkaline phosphatase (ALP) | 1.88±27.87 | 16 | -0.29±15.78 | | 14 | 0.8 | |
| Alanine aminotransferase (ALT) | -0.44±4.6 | 16 | 1.21±5.9 | | 14 | 0.4 | |
| Aspartate aminotransferase (AST) | 1.31±3.16 | 16 | 0.43±5.82 | | 14 | 0.62 | |
| Cholesterol | -4.17±12.04 | 12 | 5.38±28.19 | | 13 | 0.29 | |
| Creatine Kinase (CK) | 14.13±71.14 | 15 | 25.92±50.66 | | 13 | 0.63 | |
| Calcium (Ca) | 0.04±0.34 | 16 | 0.08±0.39 | | 14 | 0.81 | |
| Chloride (Cl) | -1.14±2.48 | 7 | 1.2±2.39 | | 5 | 0.13 | |
| Iron (Fe) | 4.79±37.65 | 15 | 16.72±53.85 | | 14 | 0.49 | |
| Glucose (Glu) | 3.31±14.46 | 16 | -9.5±22.74 | | 14 | 0.07 | |
| **Potassium (K)** | -0.09±0.39 | 15 | 0.02±0.24 | | 14 | 0.38 | |
| Lactate dehydrogenase (LDH) | 50.79±65.68 | 14 | 9.07±73.6 | | 14 | 0.12 | |
| Sodium (Na) | 0.31±2.21 | 16 | -0.21±2.04 | | 14 | 0.51 | |
| Prolactin (PRL) | -0.93±2.58 | 16 | -0.5±4.34 | | 14 | 0.74 | |
| Total protein (PROT-T) | 0.19±2.74 | **16** | 1.71±2.46 | | **14** | **0.01** | |
| Triglycerides (TG) | -26.75±47.52 | 12 | -3.08±79.37 | | 13 | 0.38 | |
| Transferrin (TRF) | -0.02±0.23 | 5 | 0.28±0.28 | | 5 | 0.1 | |
| Transferrin saturation (TRFsat) | 1.4±10.39 | 5 | 1.78±12.82 | | 5 | 0.96 | |
| Urea | 1.46±5.78 | 16 | 1.04±6.4 | | 14 | 0.85 | |
| Creatinine (CR) | 0±0.04 | 14 | 0.01±0.07 | | 14 | 0.71 | |
| Free T4 (FT4) | -0.74±1.68 | 14 | 0.11±1.22 | | 14 | 0.14 | |
| Hematocrit (HCT) | 0.06±1.36 | 13 | 1.04±2.37 | | 14 | 0.21 | |
| Platelets (PLT) | -13.46±31.38 | **13** | 29.64±26.2 | | **14** | **0.0007** | |
| Thyroid stimulating hormone (TSH) | 0.41±1.03 | 16 | 0.03±0.84 | | 14 | 0.29 | |
| White blood cells (WBC) | -0.1±1.97 | 13 | 0.55±0.97 | | 14 | 0.28 | |
| Hemoglobin (HGB) | 0.02±0.36 | 13 | 0.32±0.8 | | 14 | 0.22 | |
| Testosterone | 0±0 | 11 | -2.41±6.91 | | 11 | 0.26 | |
